# Insights of Clinical Significance From 109 695 Solid Tumor Tissue-Based Comprehensive Genomic Profiles

**DOI:** 10.1093/oncolo/oyad251

**Published:** 2023-09-08

**Authors:** Andreas M Heilmann, Jonathan W Riess, Margaret McLaughlin-Drubin, Richard S P Huang, Meghann Hjulstrom, James Creeden, Brian M Alexander, Rachel L Erlich

**Affiliations:** Foundation Medicine, Cambridge, MA, USA; University of California Davis Comprehensive Cancer Center, Sacramento, CA, USA; Foundation Medicine, Cambridge, MA, USA; Foundation Medicine, Cambridge, MA, USA; Foundation Medicine, Cambridge, MA, USA; Foundation Medicine, Cambridge, MA, USA; Foundation Medicine, Cambridge, MA, USA; Foundation Medicine, Cambridge, MA, USA

**Keywords:** comprehensive genomic profiling, solid tumors, precision medicine, in vitro diagnostic, next generation sequencing, oncology

## Abstract

**Background:**

FoundationOneCDx is approved in the US and Japan as a companion diagnostic test to identify patients with cancer who may benefit from treatment with 30 drug therapies in the US and 23 in Japan. Tumor profiling with FoundationOneCDx also detects genomic findings with evidence of clinical significance that may inform clinical care decisions beyond companion diagnostic claims. This observational study reports the breadth and impact of clinical decision insights from FoundationOneCDx solid tumor profiles.

**Materials and Methods:**

Consecutive test result reports for patients with solid tumor diagnoses (*n* = 109 695) were retrospectively analyzed for clinically significant predictive, prognostic, and diagnostic genomic alterations and signatures, determined in accordance with professional guidelines. Interventional clinical trials with targeted therapies or immune checkpoint inhibitors were matched to tumor profiles based on evidence that the genomic finding may be an actionable, investigational, or hypothetical target in the patient’s tumor type.

**Results:**

In 14 predefined cancer types (80.7% of analyzed solid tumors), predictive, prognostic, and diagnostic markers were reported in 47.6%, 13.2%, and 4.5% of samples, respectively, accounting for a total of 51.2% of tumor profiles. Pan-cancer predictive markers of tumor mutational burden (TMB) of 10 or more mutations per megabase, high microsatellite instability (MSI), or *NTRK1/2/3* fusions were observed in 15.6%, 2.0%, and 0.1% of solid tumors, respectively. Most solid tumor profiles (89.2%) had genomic results that could theoretically inform decisions on the selection of immunotherapy and targeted therapy clinical trials.

**Conclusion:**

For this real-world population of patients with FoundationOneCDx solid tumor profiles in the routine course of clinical care, clinically significant findings were reported for approximately half of patients with genomic results.

## Teaser

This retrospective study reports clinically significant findings from tumor tissue profiling with FoundationOneCDx for a large global cohort of patients with advanced solid tumors.

**Implications for Practice**

This retrospective observational study of 109 695 solid tumor profiles with FoundationOneCDx investigates the type and frequency of clinically significant genomic findings for patients with malignant solid tumors. Clinically significant insights were identified in 51.2% of tissue samples from 14 analyzed cancer types, with predictive markers detected in 47.6%, prognostic markers in 13.2%, and diagnostic markers in 4.5% of samples. Furthermore, 89.2% of tumor profiles had genomic results that could theoretically inform the selection of interventional trials. Approximately half of patients with solid tumors received clinically significant insights from comprehensive genomic tumor profiles, most of which support treatment decisions according to drug approvals or professional guidelines.

## Introduction

Comprehensive genomic profiling (CGP) tests in oncology are based on next-generation sequencing and analyze the genomic makeup of a tumor by detecting multiple types of molecular alterations (single-nucleotide variants, small insertions and deletions, copy number alterations, and genomic rearrangements, including gene fusions).^1^ These tests typically cover the entire coding regions of broad panels of cancer-related genes and aim to enable physicians to molecularly characterize their patients’ tumors and to identify patients who may or may not benefit from targeted therapies or immune checkpoint inhibitors. The presence or absence of genomic markers can also aid in the selection of non-targeted treatment approaches, including chemo- or immunotherapies or combinations thereof, and can inform the patient’s diagnosis or prognosis. If tumor tissue samples of sufficient quality or quantity are not available, CGP of liquid biopsies can be performed to inform treatment decisions, especially for patients who are not candidates for tissue biopsies or have progressed on prior targeted therapy.

FoundationOne CDx is the first broad companion diagnostic approved by the US Food and Drug Administration (FDA) in 2017 and is indicated for patients with solid tumors. It uses hybridization-based capture paired with next-generation sequencing to detect substitutions, insertions and deletions, and copy number alterations in 324 genes; select gene rearrangements are also detected, as well as the genomic signatures tumor mutational burden (TMB), microsatellite instability (MSI), and genomic loss of heterozygosity (LOH, in ovarian cancer) in DNA from formalin-fixed, paraffin-embedded (FFPE) tumor tissue samples.^2^ As of June 2022, the test is indicated as a companion diagnostic for 30 drug therapies in 7 tumor types and 3 drug therapies across solid tumor types (pan-tumor) in accordance with the approved drug labeling. There has been a steady growth of companion diagnostic markers supporting the increasing utility of precision treatments in oncology.^2^ Tumor mutation profiling with FoundationOne CDx is also intended for healthcare professionals to detect genomic findings of clinical significance beyond companion diagnostic results in accordance with professional guidelines in oncology. These findings include markers of predictive, prognostic, or diagnostic significance,^3^ as well as alterations with potential germline or clonal hematopoiesis implications. Alterations of potential clinical significance can be important to select patients for genomically matched trials or can become clinically relevant in the future, thereby supporting access to clinical trials for as many patients as possible.

This study aimed to retrospectively review the clinically significant findings from tumor tissue profiling with FoundationOne CDx for a large global cohort of patients with advanced solid tumors. Specifically, we investigated the types and frequencies of predictive, prognostic, and diagnostic markers according to drug approvals and professional guidelines in 14 cancer types and describe the frequency of pan-tumor predictive genomic markers across the cohort. We also explored the landscape of interventional clinical trials matched to the patients’ genomic profiles. This study demonstrates how CGP of tumor tissue samples paired with comprehensive interpretation and reporting impacts the identification of markers with evidence of clinical significance across solid tumor types and of investigational targets that may inform patient care.

## Materials and Methods

### Patient Population

The frequencies of therapeutic, diagnostic, and prognostic markers as well as of matched clinical trials were analyzed for patients who received FoundationOne CDx profiling reports in the course of their clinical care between April 1, 2020, and March 31, 2021. Failed, cancelled, and amended reports were excluded from these analyses. Subset analyses were performed within the population of patients with 14 predefined cancer types: non-small cell lung cancer (NSCLC; *n* = 22 152), colorectal cancer (CRC; *n* = 13 193), breast cancer (*n* = 11 016), ovarian cancer (*n* = 6999), prostate cancer (*n* = 6513), pancreatic adenocarcinoma (*n* = 6168), gastroesophageal adenocarcinoma (adenocarcinoma of the stomach, esophagus, or gastroesophageal junction; *n* = 4762), unknown primary carcinoma (carcinoma, adenocarcinoma, squamous cell carcinoma, or unspecific malignant neoplasm of unknown primary; *n* = 4607), urothelial carcinoma (*n* = 3236), cholangiocarcinoma (intrahepatic and extrahepatic; *n* = 2901), melanoma (cutaneous, mucosal, or not specified; *n* = 2743), glioma (*n* = 2350), head and neck squamous cell carcinoma (*n* = 1787), and uveal melanoma (*n* = 142). Approval for this study, including a waiver of informed consent and a HIPAA waiver of authorization, was obtained from the Western Institutional Review Board (Protocol No. 20152817).

### FoundationOne CDx assay and clinical reporting

FoundationOne CDx laboratory analysis was performed as previously described.^2^ Bioinformatic analysis is based on an in-house analysis pipeline that detects 4 types of alterations: substitutions, insertions and deletions, rearrangements, and copy number alterations. Custom in-house software applications are used to review and annotate genomic alterations and signatures, including TMB and MSI across tumor types and LOH in ovarian cancer. Variant calling and categorization are continuously monitored and refined based on external and internal evidence according to standard operating procedures.

For report generation, Foundation Medicine utilizes National Comprehensive Cancer Network (NCCN) guidelines and compendia; guidelines by the American Society of Clinical Oncology (ASCO), European Society of Medical Oncology (ESMO), or World Health Organization (WHO); scientific and medical journal subscriptions; public and paid access to conference resources; and PubMed, ClinVar, and Catalogue of Somatic Mutations in Cancer (COSMIC) databases. Content and report generation are both managed by in-house proprietary software. As new evidence becomes publicly available, the content is continually updated in real time according to predefined reporting criteria and standards.

### Markers of Clinical Significance

**Figure 1.**
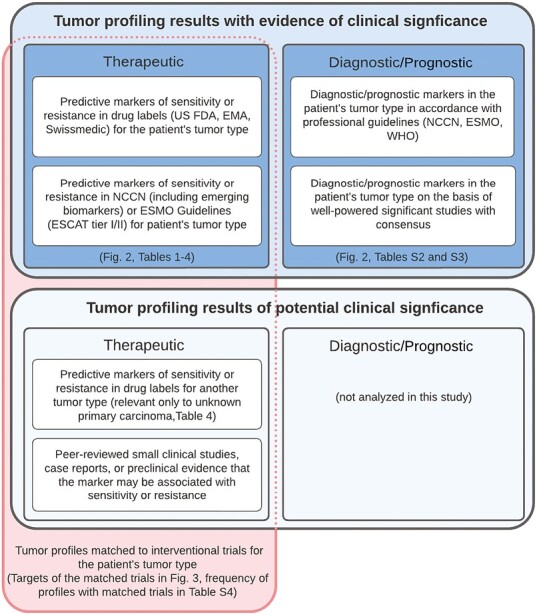
Criteria for the markers with evidence of clinical significance and the matching of clinical trials to tumor profiles with actionable, investigational, or hypothetical therapeutic markers. Categories and tiers for the interpretation of the clinical significance of genomic alterations and signatures in tumor profiles were adapted from the Association for Molecular Pathology/American Society of Clinical Oncology/College of American Pathologists Guidelines.^3^ The criteria for evidence of therapeutic significance were modified to include predictive markers in drug labels (US FDA, EMA, Swissmedic; eg, *BRAF* V600E mutation in non-small cell lung cancer, colorectal cancer, or melanoma) or professional guidelines (NCCN and ESMO Guidelines; eg, *ERBB2* amplification in colorectal cancer) that are used to select treatments in the patient’s tumor type, with ESCAT levels of evidence tiers I and II being considered actionable.^4^ These therapeutic markers are detailed in Tables 1-4. Diagnostic markers inform the diagnosis, and prognostic markers inform the prognosis, for the patient’s tumor type based on evidence in professional guidelines or from well-powered significant clinical studies with consensus in the literature. The prognostic (eg, *KRAS* G12V mutation in non-small cell lung cancer) or diagnostic markers (eg, *IDH1* R132C mutation in glioma) are listed in Supplementary Tables S2 and S3, respectively. The frequency of the tumor profiling results with evidence of clinical significance is summarized by cancer type and marker category in Fig. 2. Therapeutic markers with evidence of clinical significance or of potential significance were matched to interventional trials for the patient’s tumor type and profile and ranked as described in the Methods section. The targets of the therapies in the matched and ranked trials on the tumor profiling reports are analyzed in Fig. 3. The frequency of tumor profiles with trial matches is provided by cancer type in Supplementary Table S4.

Predictive markers were defined as therapeutically relevant markers in FDA, European Medicines Agency (EMA), or Swissmedic drug labels or NCCN guidelines or targets of ESMO Scale for Clinical Actionability of molecular Targets (ESCAT) evidence tier I/II.^4-6^ Prognostic and diagnostic markers were determined in accordance with NCCN, ESMO, or WHO professional guidelines or, in select instances, supported by well-powered studies with consensus in the literature (Fig. 1). Each FoundationOne CDx sample is reviewed by a licensed pathologist for quality purposes and assigned a disease ontology that is consistent with the diagnosis stated by the ordering physician. The disease ontologies are categorized as cancer types according to WHO guidelines and annotated to accurately reflect the disease and histopathology in drug labels or professional guidelines irrespective of disease stage or line of treatment. Relevant alterations were identified based on the specific variants (eg, *BRAF* V600E, and *ERBB2* amplification), curated functional or likely functional status (eg, known or likely activating mutations in *PIK3CA*, rearrangements in oncogenes), or events that may indicate the disruption of tumor suppressor genes (eg, *BRCA1/2* rearrangements, partial losses, truncating variants, or germline variants in ClinVar). Genomic signatures were called according to Foundation Medicine’s published and validated algorithms for TMB score and MSI status, or for genomic LOH score in ovarian cancer samples.^2,7-9^

**Table 1.** Predictive markers and their frequencies in clinical reports for patients with non-small cell lung cancer.

Predictive marker	Frequency	Percentage
*N* (all non-small cell lung cancer reports)	22 152	100.0
TMB ≥ 10 mut/Mb	7055	31.8
*EGFR* mutations	3263	14.7
* EGFR* exon 19 deletion	1600	7.2
* EGFR* exon 21 L858R mutation	1078	4.9
* EGFR* uncommon sensitizing mutation	335	1.5
* EGFR* G719X/S768I/L861Q mutation	282	
* EGFR* E709X/E709_T710 > D/L861R mutation^1^	49	
* EGFR* exon 19 insertion^1^	12	
* EGFR* exon 20 insertion	277	1.3
* EGFR* exon 20 FQEA insertion	18	
* EGFR* T790M mutation	211	1.0
* EGFR* T790M and exon 19 deletion	112	
* EGFR* T790M and exon 21 L858R mutation	79	
* EGFR* T790M and other *EGFR* mutation	14	
*KRAS* G12C mutation	2331	10.5
*MET* alteration	1186	5.4
* MET* focal amplification^1^	712	3.2
* MET* focal amplification and *EGFR* mutation^1^	156	
* MET* exon 14 alteration	527	2.4
* MET* exon 14 alteration and *MET* amplification	53	
*ALK* fusion/rearrangement	669	3.0
*ERBB2* activating mutation^1^	476	2.1
*BRAF* V600E mutation	344	1.6
*RET* fusion/rearrangement	222	1.0
*ROS1* fusion/rearrangement	208	0.9
MSI high	81	0.4
*NTRK1/2/3* fusion	29	0.1

^1^Markers in professional guidelines only at time of analysis.

**Table 2.** Predictive markers and their frequencies in clinical reports for patients with common gastrointestinal cancers.

Cancer type	Predictive marker	Frequency	Percentage
Colorectal cancer		13 193	100.0
	*KRAS* activating mutation	6409	48.6
	* KRAS* G12C mutation	445	3.4
	TMB ≥ 10 mut/Mb	1254	9.5
	*BRAF* V600E mutation	969	7.3
	MSI high	661	5.0
	*NRAS* activating mutation	564	4.3
	*ERBB2* amplification^1^	410	3.1
	* ERBB2* amplification and no * BRAF/KRAS/NRAS* activating mutation^1^	328	2.5
	*NTRK1/2/3* fusion	24	0.2
Pancreatic adenocarcinoma		6168	100.0
	*BRCA* alteration	231	3.7
	* BRCA2* alteration	167	2.7
	* BRCA1* alteration	65	1.1
	TMB ≥ 10 mut/Mb	94	1.5
	*PALB2* alteration^1^	38	0.6
	MSI high	30	0.5
	*NTRK1/2/3* fusion	4	0.1
Gastroesophageal adenocarcinoma		4762	100.0
	*ERBB2* amplification	714	15.0
	TMB ≥ 10 mut/Mb	445	9.3
	MSI high	159	3.3
	*NTRK1/2/3* fusion	5	0.1
Cholangiocarcinoma		2901	100.0
	IDH1 R132 mutation	324	11.2
	*FGFR2* fusion/rearrangement	235	8.1
	TMB ≥ 10 mut/Mb	123	4.2
	*BRAF* V600E mutation^1^	52	1.8
	MSI high	40	1.4
	*NTRK1/2/3* fusion	2	0.1

^1^Markers in professional guidelines only at time of analysis.

**Table 3.** Frequencies of predictive markers in clinical reports for patients with breast, ovarian, prostate, or urothelial cancer.

Cancer type	Predictive marker	Frequency	Percentage
Breast cancer		11 016	100.0
	*PIK3CA* mutation	4082	37.1
	* PIK3CA* mutation and no *ERBB2* amplification	3779	
	*ESR1* mutation^1^	1134	10.3
	*BRCA* alteration	1032	9.4
	* BRCA2* alteration	608	5.5
	* BRCA2* alteration and no *ERBB2* amplification	587	
	* BRCA1* alteration	438	4.0
	* BRCA1* alteration and no *ERBB2* amplification	423	
	TMB ≥ 10 mut/Mb	1010	9.2
	*ERBB2* amplification	832	7.6
	MSI high	40	0.4
	*NTRK1/2/3* fusion	15	0.1
Ovarian cancer		6999	100.0
	Genomic LOH ≥ 16%	2150	30.7
	*BRCA* alteration	969	13.8
	* BRCA1* alteration	648	9.3
	* BRCA2* alteration	327	4.7
	TMB ≥ 10 mut/Mb	280	4.0
	MSI high	56	0.8
	*NTRK1/2/3* fusion	9	0.1
Prostate cancer		6513	100.0
	*PTEN* alteration^1^	1772	27.2
	* PTEN* homozygous loss (all or select exons)^1^	1182	18.1
	Non-*BRCA1/2* HRR gene alteration	1194	18.3
	* CDK12* alteration	550	8.4
	* ATM* alteration	377	5.8
	* CHEK2* alteration	109	1.7
	* PALB2* alteration	47	0.7
	* BRIP1* alteration	33	0.5
	* BARD1* alteration	27	0.4
	* RAD54L* alteration	25	0.4
	* RAD51B* alteration	23	0.4
	* RAD51C* alteration	23	0.4
	* RAD51D* alteration	16	0.2
	* FANCL* alteration	14	0.2
	* CHEK1* alteration	7	0.1
	*BRCA* alteration	660	10.1
	* BRCA2* alteration	587	9.0
	* BRCA1* alteration	80	1.2
	TMB ≥ 10 mut/Mb	339	5.2
	MSI high	173	2.7
	*NTRK1/2/3* fusion	4	0.1
Urothelial carcinoma		3236	100.0
	TMB ≥ 10 mut/Mb	1119	34.6
	*FGFR2/3* alterations	560	17.3
	* FGFR3* activating mutation	474	14.6
	* FGFR3* fusion/rearrangement	86	2.7
	* FGFR2* fusion/rearrangement	10	0.3
	MSI high	30	0.9
	*NTRK1/2/3* fusion	5	0.2

^1^Markers in professional guidelines only at time of analysis.

**Table 4.** Predictive markers and their frequencies in clinical reports for patients with solid tumors, melanoma, glioma, head and neck squamous cell carcinoma, and unknown primary carcinoma.

Cancer type	Predictive marker	Frequency	Percentage
Solid tumors		109 503	100.0
	TMB ≥ 10 mut/Mb	17 056	15.6
	MSI high	2149	2.0
	*NTRK1/2/3* fusion	141	0.1
Melanoma		2743	100.0
	TMB ≥ 10 mut/Mb	1435	52.3
	*BRAF* V600X mutation	856	31.2
	*NRAS* mutation^1^	660	24.1
	*KIT* mutation^1^	147	5.4
	*NTRK1/2/3* fusion	5	0.2
	MSI high	4	0.1
Glioma		2350	100.0
	*IDH1* R132 mutation^1^	446	19.0
	*BRAF* V600E mutation^1^	80	3.4
	*BRAF* fusion^1^	66	2.8
	*NTRK1/2/3* fusion	6	0.3
Head neck squamous cell carcinoma		1787	100.0
	*CDKN2A* alteration^1^	570	31.9
	TMB ≥ 10 mut/Mb	326	18.2
	*EGFR* amplification^1^	127	7.1
	*HRAS* mutation^1^	72	4.0
	MSI high	13	0.7
	*NTRK1/2/3* fusion	0	0.0
Unknown primary carcinoma^2^		4607	100.0
	TMB ≥ 10 mut/Mb	934	20.3
	*BRCA* alteration	235	5.1
	* BRCA2* alteration	134	2.9
	* BRCA1* alteration	103	2.2
	*ERBB2* amplification	171	3.7
	*KRAS* G12C mutation	170	3.7
	*BRAF* V600E mutation	123	2.7
	MSI high	118	2.6
	*FGFR2* fusion/rearrangement	99	2.1
	*EGFR* mutations	47	1.0
	*ALK* fusion/rearrangement	18	0.4
	*RET* fusion/rearrangement	10	0.2
	*MET* exon 14 alteration	9	0.2
	*ROS1* fusion/rearrangement	7	0.2
	*NTRK1/2/3* fusion	1	<0.1

^1^Markers in professional guidelines only at time of analysis.

^2^Predictive markers in unknown primary carcinoma include predictive pan-cancer markers (TMB ≥ 10 mut/Mb, MSI-high, and *NTRK1/2/3* fusion) as well as investigational markers that are associated with therapy approvals in other tumor types at time of analysis.

### Clinical Trial Reporting and Analyses

Interventional trials with targeted therapies or immune checkpoint inhibitors were matched to each clinical report according to the patient’s specific genomic alterations (with exclusion for resistance), tumor type, and age. To be included as a trial, the therapy or an agent of the same class must have demonstrated clinical activity for a genomic alteration that is similar to the patient’s alteration or at least have strong preclinical evidence that a patient with a similar alteration may respond to the therapy (Fig. 1). Trials were not matched to markers of potential clinical significance that are not considered actionable in the patient’s tumor type (eg, single-agent BRAF inhibitor trials were not reported in association with *BRAF* V600E mutation in CRC). Matched clinical trials were ordered by gene or genomic signature and prioritized based on age range inclusion criteria for patients in pediatric care, proximity to ordering medical facility, trial phase (favoring later phases), and whether trial information has been verified within the last 2 months. For each gene or genomic signature, the 10 highest-priority matched trials were included on clinical reports and analyzed for their targets.

## Results

**Figure 2.**
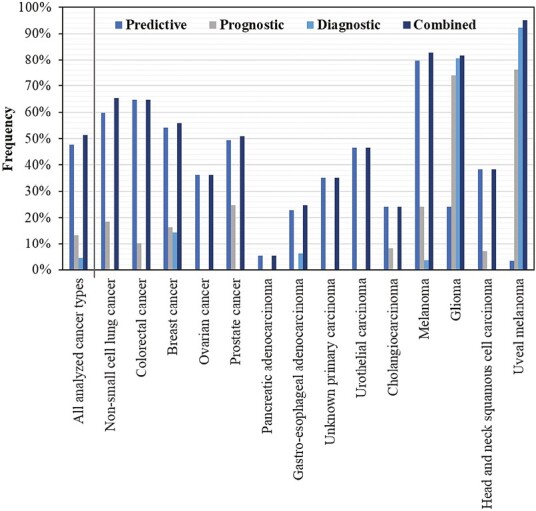
Relative frequency of clinically significant predictive, prognostic, and diagnostic markers and their combination in the profiled solid tumors. All 14 analyzed cancer types (*n* = 88 569) include non-small cell lung cancer (*n* = 22 152), colorectal cancer (*n* = 13 193), breast cancer (*n* = 11 016), ovarian cancer (*n* = 6999), prostate cancer (*n* = 6513), pancreatic adenocarcinoma (*n* = 6168), gastroesophageal adenocarcinoma (*n* = 4762), unknown primary carcinoma (*n* = 4607), urothelial carcinoma (*n* = 3236), cholangiocarcinoma (*n* = 2901), melanoma (*n* = 2743), glioma (*n* = 2350), head and neck squamous cell carcinoma (*n* = 1787), and uveal melanoma (*n* = 142). Note that the markers in unknown primary carcinoma are predictive pan-cancer markers (TMB ≥ 10 mut/Mb, MSI-high, and *NTRK1/2/3* fusion) as well as investigational targets that are associated with therapy approvals in other tumor types.

Between April 1, 2020, and March 31, 2021, 109 695 clinical reports were generated based on FoundationOne CDx tumor profiles, almost all of which (99.8%) were for patients with solid tumors. Among patients with available information, 52.5% were female, and the median age was 67 (range 1-89+), with most of the patients being 65-79 (45.1%) or 40-64 (39.2%) years of age. Fourteen distinct and common cancer types were predefined for further analysis, which account for a total 80.7% of the dataset (Supplementary Table S1). Markers with evidence of clinical significance were categorized into predictive, prognostic, and diagnostic findings and determined by cancer type as described in the methods (Fig. 1). In the predefined cancer types, clinically significant predictive markers were observed in 47.6% (range 3.5%-79.7), prognostic markers in 13.2% (range 0-76.1%), and diagnostic markers in 4.5% (range 0%-92.3) of tumor samples (Fig. 2). Across the marker categories and analyzed cancer types, clinically significant findings (ie, genomic markers in oncology drug labels or professional guidelines) were reported in 51.2% (range 5.5%-95.1%) of the tumor profiles. This number is smaller than the sum of the predictive, prognostic, and diagnostic finding frequencies, as markers partially overlap between these categories. In the following paragraphs, we will summarize the findings with evidence of clinical significance by disease area and describe investigational targets for the enrollment of patients into clinical trials.

As predictive markers in NSCLC, TMB of at least 10 mutations per megabase (≥10 mut/Mb) was identified in 31.8%, sensitizing *EGFR* mutations in 15.1%, *KRAS* G12C mutations in 10.5%, *MET* alterations in 5.4% (exon 14 in 2.4%, focal amplification in 3.2%), and actionable oncogenic fusions in ~5% (*ALK* 3.0%, *RET* 1.0%, *ROS1* 0.9%, *NTRK1/2/3* 0.1%) of reports (Table 1). The *EGFR* G719X/S768I/L861Q mutations in the afatinib therapy label and NCCN guidelines made up most of the uncommon sensitizing *EGFR* mutations (1.9%), whereas rare sensitizing mutations (*EGFR* E709X, E709_T710 > D, L861R, exon 20 FQEA insertion, or exon 19 insertions) were detected in 80 samples (0.4%). Most *EGFR* T790M mutations occurred in the context of known activating *EGFR* mutations (205/211, 97.2%), in line with their established role as acquired resistance alterations.^10,11^ Concurrent pathogenic *MET* amplification and *EGFR* mutation were observed in 156 samples (0.7%, 4.8% of *EGFR*-mutated samples).^12,13^

In CRC profiles, resistance mutations that predict lack of benefit from EGFR antibodies alone or in combination with chemotherapies were frequently detected in *KRAS* (48.6%), *BRAF* (7.3%), or *NRAS* (4.3%) (Table 2). *BRAF* V600E mutations or MSI-high status (5.0%) may inform treatment with approved therapies. *ERBB2* amplification (3.1%), especially in the absence of *BRAF/KRAS/NRAS* activating mutation (2.5%), is an actionable marker for HER2-targeted treatment approaches.^14-16^ Approximately 6% of pancreatic adenocarcinoma reports identified established predictive markers (inactivating *BRCA1/2* alteration 3.7%, TMB ≥ 10 mut/Mb 1.5%, inactivating *PALB2* alteration 0.6%, MSI-high 0.5%, and *NTRK1/2/3* fusion 0.1%). *ERBB2* amplification was the most common predictive marker in gastroesophageal adenocarcinoma, and recent targeted therapy approvals and guideline updates had a significant impact for patients with cholangiocarcinoma and actionable findings in *IDH1* (11.2%), *FGFR2* (8.1%), or *BRAF* (1.8%) (Table 2).

Standard-of-care predictive markers in breast cancer include activating *PIK3CA* mutations (in hormone-receptor-positive metastatic breast cancer), which were reported in 37.1% of samples, with most (3779/4082, 92.6%) occurring in the absence of *ERBB2* amplification; other predictive markers include inactivating *BRCA1/2* alterations (9.4%) and *ERBB2* amplification (7.6%) (Table 3). *ESR1* mutations (10.3%) are emerging biomarkers with therapeutic significance in predicting resistance to aromatase inhibitors and informing treatment with novel endocrine therapies, such as elacestrant.^17,18^ In ovarian cancer, a high genomic LOH score (≥16%), which is a genomic signature of homologous recombination repair deficiency (HRD),^19^ was observed in 30.7%, inactivating *BRCA1/2* alterations in 13.8%, and other predictive markers combined in ~5% of samples. Prostate cancer reports also had a relatively high frequency of inactivating alterations in *BRCA1/2* (10.1%) or other homologous recombination repair genes (18.3%). Known or likely *PTEN* alterations were identified in 27.2% of prostate cancer profiles.^20^ The most common predictive markers in urothelial carcinoma were TMB ≥ 10 mut/Mb (34.6%), which may inform treatment with immune checkpoint inhibitors, and sensitizing alterations in *FGFR2* or *FGFR3* (17.3%), as specified in the label of the approved FGFR tyrosine kinase inhibitor erdafitinib (Table 3).^21^

The frequency of predictive marker findings of clinical significance was also assessed in reports for patients with melanoma, glioma, and head and neck squamous cell carcinoma (Table 4). In melanoma, *BRAF* V600 mutations (31.2%) are used to select patients for approved BRAF/MEK-targeted treatments, and *NRAS* (24.1%) or *KIT* (5.4%) mutations are markers of potential therapeutic significance in NCCN guidelines.^22,23^ For patients with advanced head and neck squamous cell carcinoma, inactivating *CDKN2A* alterations (ESCAT-IIA, 31.9%), *EGFR* amplification (ESCAT-IIA, 7.1%), and activating *HRAS* mutations (ESCAT-IB, 4.0%) were categorized as predictive markers on the basis of ESMO guidelines.^6^ Among the specific cancer types surveyed, glioma samples had a comparatively high relative frequency of *NTRK1/2/3* fusions (6/2350, 0.3%). Reports for patients with gliomas also identified predictive alterations in *IDH1* (19.0%) or *BRAF* (V600E mutation 3.4%, fusion 2.8%). Across solid tumors, TMB ≥ 10 mut/Mb, MSI-high status, or *NTRK1/2/3* fusions were reported for 15.6%, 2.0%, and 0.1% of samples, respectively (Table 4). On the basis of the tissue-agnostic therapy approvals for these markers, they are also predictive in unknown primary carcinoma, where they were observed in 20.3%, 2.6%, and 0.02% of samples, respectively. Reports for patients with unknown primary carcinoma were analyzed for pan-cancer predictive markers and investigational targets that may be therapeutically actionable on the basis of therapy approvals in other specific tumor types (Table 4).

Prognostic markers (criteria in Fig. 1) are provided with their frequency (Supplementary Table S2). Although prognostic alterations in CRC, breast cancer, cholangiocarcinoma, melanoma, and head and neck squamous carcinoma are also predictive for benefit from targeted therapies, alterations in NSCLC (activating *KRAS* mutations, 18.4%), prostate cancer (alterations in at least 2 of *PTEN*, *RB1*, and *TP53*, 20.4%), glioma (*TERT* promoter 56.1%, *TP53* 44.9%, *IDH1* 19.0%, *ATRX* 16.1%, *H3F3A* 4.3%, *IDH2* 0.2%), or uveal melanoma (*BAP1* 59.2%, *SF3B1* 23.9%) are specifically prognostic. Alterations in 2 or more of the *PTEN*, *RB1*, and *TP53* tumor suppressor genes may identify patients with prostate cancer of an aggressive variant (AVPC),^24^ who may benefit from intensified platinum-containing chemotherapy for metastatic castration-resistant prostate cancer.^25^

Diagnostic markers (criteria in Fig. 1) may inform the diagnosis of a histologic or molecular tumor subtype (Supplementary Table S3). Molecular markers are especially important for the molecular classification of glioma subtypes.^26^*IDH* mutations diagnostic for WHO-recognized diffuse glioma disease ontologies were detected in 19.0% (*IDH1*) and 0.2% (*IDH2*) of gliomas. Together with *IDH* mutation, alterations in *ATRX* (16.1%) and *TP53* (44.9%) are characteristic of diffuse astrocytoma, *IDH* mutant,^26^ with concurrent *CDKN2A/B* loss indicating WHO grade 4 astrocytoma.^27^*EGFR* amplification (23.1%) is enriched in *IDH*-wildtype glioblastoma. *TERT* promoter mutations (56.1%) are frequent in glioblastoma or, when concurrent with *IDH* mutation and 1p/19q co-deletion, which is not clinically available on FoundationOne CDx, point to oligodendroglioma. K27M mutations in histone H3 genes, most commonly in *H3-3A* (4.3% of glioma samples), indicate diffuse midline glioma, *H3-3A* G34R mutations (1.4% of samples) hemispheric glioma,^27^ and *BRAF* fusions (2.8%) pilocytic astrocytoma.^28^ In other cancer types, *CDH1* alterations are characteristic of lobular breast cancer^29^ or hereditary diffuse-type gastric cancer.^30^*GNA11* or *GNAQ* hotspot mutations may be of diagnostic value for uveal (49.3% and 44.4%, respectively) or blue nevi-related melanomas (1.8% of melanomas each).^31,32^

**Figure 3.**
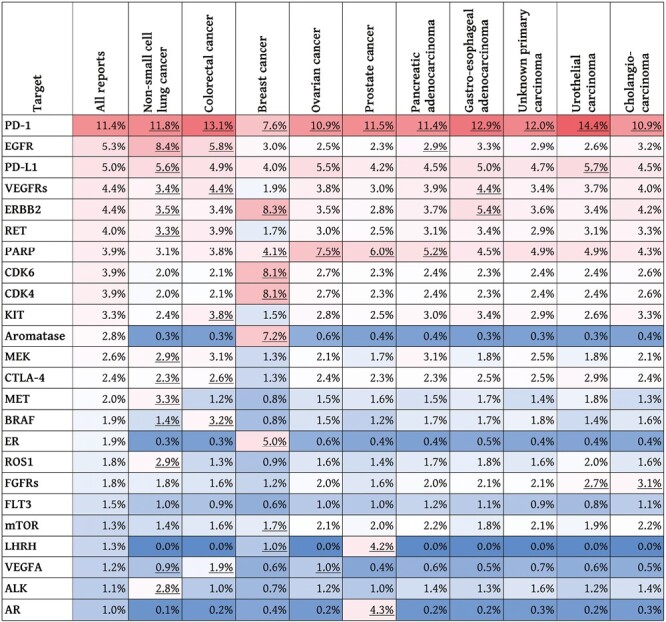
Relative frequency of the targets of therapies in interventional clinical trials matched to 109 695 clinical reports based on the patient’s tumor type, age, and evidence that the genomic finding is an actionable, investigational, or hypothetical marker (criteria in Fig. 1). The figure describes the landscape of therapy targets accounting for at least 1% of the targets in matched trials by ranking their relative frequency across all reports and showing their frequency in 10 select cancer types. For example, PD-1, PD-L1, or CTLA-4 indicate how many of the matched clinical trials evaluate immunotherapies against these targets, whereas aromatase, ER, LHRH, or AR point to hormonal agents under clinical investigation. Color shading indicates frequency in each column, with red being more and blue less frequent. The frequency of a target can depend on various distinct genomic markers; for example, trials targeting PARP are matched to alterations in different homologous recombination repair genes, such as *BRCA1, BRCA2, ATM, BRIP1, CHEK2*, etc. Targets of therapies approved in the cancer types are underlined, irrespective of genomic alterations or biomarkers in the drug label (eg, PD-1 and CTLA-4 are approved targets due to the nivolumab plus ipilimumab indication in MSI-high or mismatch repair-deficient colorectal cancer).

Tumor profiling results may inform the enrollment of patients with cancer into clinical trials. FoundationOne CDx reports match interventional trials with targeted therapies and immune checkpoint inhibitors curated from clinicaltrials.gov to the patient’s tumor profile, disease ontology, and age based on peer-reviewed evidence that the identified genomic finding is potentially actionable (Fig. 1). During the study period, information on genomically matched clinical trials was provided on most reports (89.2%) (Supplementary Table S4), ranging from 81.8% in prostate cancer to 98.7% in pancreatic adenocarcinoma, when analyzed by the 10 most frequent predefined cancer types. Most of the trial matches are due to evidence-based associations between actionable, investigational, or hypothetical genomic alterations and targeted agents, as there are limited genomic positive predictive markers for immunotherapy beyond the genomic signatures of TMB ≥ 10 mut/Mb and MSI-high, which were seen in combined 15.6% of all solid tumor samples (Table 4). To investigate the diversity of targeted approaches in interventional studies across these cancer types, we evaluated the frequency of experimental targets of the matched clinical trials. The most common target was the immune checkpoint receptor programmed cell death protein 1 (PD-1; 11.4%), followed by *EGFR* (5.3%) and the PD-1 ligand PD-L1 (5.0%) (Fig. 3), with immunotherapy targets accounting for approximately 22% (data not shown) of all targets in matched trials. Certain targets were enriched in specific cancer types as expected based on the clinical treatment landscape or tumor biology (eg, EGFR, MET, ROS1, MET, and ALK in NSCLC; or ERBB2, CDK4, CDK6, aromatase, and ER in breast cancer). The targets of therapies approved in the respective cancer types (underlined numbers in Fig. 3) suggest that the matched trials provide investigational options for a wide variety of targets that are currently not actionable with approved treatments and highlight the potential of clinical development across different cancer types.

## Discussion

We present clinical decision insights from 109695 FoundationOne CDx clinical reports. Although most samples were from patients in the US, a significant portion of samples came from patients outside of the US (~11% from Asia-Pacific, 5% from Europe/Middle East/Africa, and 5% from Central/South America). The observed distribution of diseases follows both the prevalence of tumor types among patients with advanced cancers and the availability of targeted treatments that require the detection of genomic markers. Unknown primary carcinoma samples accounted for 4.2% of profiled samples, some of which may have been refined in their diagnosis after submission for FoundationOne CDx.^33^ As systemic treatment options and guideline-recommended markers beyond MSI-high, TMB ≥ 10 mut/Mb, and *NTRK* fusions are limited in unknown primary carcinoma, we opted to analyze these samples for the frequency of markers that are companion diagnostics in at least 1 solid tumor indication. These markers are under active clinical investigation in the CUPISCO trial for advanced unknown primary carcinoma^34^ and combined accounted for more than 40% of reports, with TMB ≥ 10 mut/Mb in 20.3%, inactivating *BRCA* alterations in 5.1%, *ERBB2* amplification in 3.7%, and *KRAS* G12C mutation in 3.7% of samples (Table 4). Of note, several of these alterations are established markers to guide treatment decisions in multiple disparate cancer types. Other findings, such as *FGFR2* fusions, *EGFR* mutations, or *MET* exon 14 alterations, may also help refine diagnoses, as they are highly enriched in specific cancer types (Tables 1 and 2).

Tumor profiling insights on the status of multiple disease-relevant genes can inform treatment decisions on the initiation, discontinuation, or combination of targeted therapies, as illustrated in NSCLC and CRC: 1) *EGFR*-mutated lung cancer is one of the best studied contexts of primary or secondary (acquired) resistance mechanisms in solid tumors (Supplementary Table S5).^11^ Within the NSCLC profiles with sensitizing *EGFR* mutations in this dataset (*n* = 3260), which were not selected for stage or prior treatment with EGFR inhibitor, potential on-target *EGFR* resistance mutations were identified in 7.9% of samples. Potential off-target resistance through established bypass signaling mechanisms were detected in 12.1% of samples (*MET* amplification 4.8%, *BRAF/MEK/RAS* activation 4.1%, *ERBB2* activation 2.7%, or oncogenic fusions 1.0%). Furthermore, 9.7% of *EGFR*-mutated NSCLC samples harbored concurrent *RB1* and *TP53* alterations, which are known to predict worse outcomes on EGFR inhibitors and an increased risk of histologic transformation to small cell lung cancer.^35-37^ Moreover, a validated test of sufficient tissue specimen that reports pertinent negative results for the disease-relevant actionable drivers offers important clinical decision support regarding targeted therapy or immunotherapy for metastatic, locally advanced, and early stage NSCLC.^38-44^ 2) In metastatic CRC, *ERBB2* amplification is an emerging marker that may predict response to HER2-targeted agents^14-16^; however, several studies report that patients with concurrent *BRAF*, *KRAS*, or *NRAS* derive less benefit from HER2-targeted therapies. Regardless of *BRAF*, *KRAS*, or *NRAS* mutation status, *ERBB2* amplification is also associated with inferior outcomes for patients with metastatic CRC treated with EGFR antibodies.^45,46^ In conclusion, alterations in all 4 of these genes need to be considered for decisions on treatment with approved EGFR antibodies or enrollment into clinical trials with HER2-targeted agents in CRC.

Tumor CGP regularly results in secondary genomic findings, including potential pathogenic germline variants and variants that may be associated with clonal hematopoiesis. FoundationOne CDx reports highlight pathogenic or likely pathogenic variants with a variant allele frequency of over 10% in 24 cancer susceptibility genes^47^ as potential pathogenic germline variants to support physicians and their patients in decisions about follow-up germline testing and genetic counseling. Potential pathogenic germline variants were reported in approximately 10% of FoundationOne CDx samples in a previous analysis.^48^ FoundationOne CDx reports designate specific alteration types in 14 genes known to be associated with clonal hematopoiesis.^49,50^ As these alterations may be derived from hematopoietic stem cell clones instead of the profiled tumor and may have clinical implications,^51^ they are being highlighted to the ordering physician for clinical correlation and potential further hematological workup and have been observed in 10% of tissue samples from common solid tumors.^52^ When appropriately reported to the clinical care team, secondary tumor profiling findings with potential germline or clonal hematopoiesis implications can support clinical care decisions beyond the selection of treatments of solid tumors.

This study has several limitations: 1) The cohort of patients with solid tumors profiled is subject to selection bias. This includes but is not limited to bias toward tumor types that are expected to have higher frequency of actionable markers; patients, centers, or regions with access to FoundationOne CDx; patients who are candidates for precision treatments or clinical trials; or patients with prior negative single-marker tests. 2) Although each sample has been reviewed by a pathologist before tumor profiling, limited prior workup and accompanying information may affect the tumor type diagnosis available for reporting. Due to the size of the dataset, central confirmation of the diagnosis was not feasible. Therefore, the marker frequencies described here may not reflect the final diagnosis for all patients tested. 3) The genomic findings reported by FoundationOne CDx need to be contextualized for any individual patient with each patient’s history, clinical variables (eg, tumor stage, pathological diagnosis, performance status), and treatment goals. The predictive markers in this study are currently mostly relevant for patients with advanced disease; some markers require additional pathological information (eg, hormone receptor status for *PIK3CA* mutations in breast cancer), and other findings can guide later-line treatment options after prior standard-of-care therapy. 4) The tumor profiles reported in this study came from primary and metastatic tumors and were not selected or stratified for the patients’ treatment history. Samples from patients who were previously treated, especially after prior targeted therapy, may be more genomically complex due to evolution of tumors under treatment selection. Therefore, we cannot conclude whether the alterations observed concurrently with sensitizing *EGFR* mutations in NSCLC (Supplementary Table S5) were mediating primary or acquired resistance to EGFR inhibitors. Their interpretation as markers of potential therapeutic significance is based solely on evidence of resistance to EGFR inhibitors in the literature and requires clinical correlation by the treating physician. 5) This analysis included predictive markers supported by recent clinical utility studies (eg, *KRAS* G12C mutation in NSCLC) that may not be of routine use worldwide due to limited drug availability or reimbursement. Since the analysis of this dataset in 2021, 2 accelerated FDA approvals of targeted therapies in 2022 and the respective supporting studies elevated the status of *RET* fusions and *BRAF* V600E mutation as actionable therapeutic markers with evidence of clinical significance across advanced solid tumors.^53^ Emerging biomarkers (eg, *HRAS* mutation in head and neck squamous cell carcinoma) may be actionable with only investigational approaches that are not approved yet. 6) The therapeutic markers used for clinical trial matching span a range of levels of evidence, as described in Fig. 1, from predictive markers of clinical significance (eg, *EGFR* L858R mutation in NSCLC) to hypothetical markers supported by case reports or strong preclinical evidence (eg, *MAP2K1* amplification in breast cancer).^54^ Therefore, confidence in the actionability of the markers with clinical trial matches varies depending on the supporting evidence, which is summarized in the clinical report content. In addition to the range in the actionability of the target, expected efficacy and toxicity of the matched investigational therapeutic approaches also vary and need to be contextualized for the individual patient.

The emerging, investigational, and hypothetical markers emphasize the need for ongoing clinical development, including for rare actionable alterations or cancer types. The high fraction of clinical reports with evidence-matched targeted trials highlights the potential to identify patients with predictive markers that are established today or investigational markers that may be considered for the selection of clinical trials. Although there may be variability in the relevance of the of genomic aberrations identified in patient tumors and the potential efficacy of the matched targeted therapy or immunotherapy, personalized targeted therapies have been shown to improve clinical outcomes compared to cytotoxic chemotherapy and non-personalized targeted therapies in clinical trials.^55,56^ With the genomic information provided, the treating physician can better contextualize the genomic results with clinical variables, tumor stage, and potential activity of the targeted agent or immunotherapy to identify the best next treatment—whether it be clinical trials or standard of care. By ensuring that all patients are tested for known and emerging cancer biomarkers, CGP advances cancer care to a state in which each patient’s treatment options are informed by the genomic profile of their disease.

## Supplementary Material

oyad251_suppl_Supplementary_Tables

## Data Availability

All data relevant to the study are included in the article or uploaded as supplementary information.

## Previous Presentation

The study was presented as an abstract at the 2022 Annual Meeting of the American Society of Clinical Oncology.^57^
