# Supplementary material for: Insights of Clinical Significance From 109 695 Solid Tumor Tissue-Based Comprehensive Genomic Profiles
Source: Oncologist. 2023 Sep 8;29(2):e224–36. doi: 10.1093/oncolo/oyad251 (PMC10836312; doi:10.1093/oncolo/oyad251)
Supplement: oyad251_suppl_Supplementary_Tables [file oyad251_suppl_supplementary_tables.docx]

**SUPPLEMENTARY MATERIAL
Supplementary Tables

Table S1. Dataset characteristics: Absolute and relative frequencies of tumor types and the distribution of sex and age among the patients who received FoundationOne®CDx testing during the study period.**

| **Category** | **Characteristic** | **Frequency** | **Percentage** |
| --- | --- | --- | --- |
| N (all tumor samples analyzed) | | 109695 | 100.0% |
| Cancer type | Solid tumors | 109503 | 99.8% |
|  | Select common cancer types | 88569 | 80.7% |
|  | Non-small cell lung cancer | 22152 | 20.2% |
|  | Colorectal cancer | 13193 | 12.0% |
|  | Breast cancer | 11016 | 10.0% |
|  | Ovarian cancer | 6999 | 6.4% |
|  | Prostate cancer | 6513 | 5.9% |
|  | Pancreatic adenocarcinoma | 6168 | 5.6% |
|  | Gastroesophageal adenocarcinoma | 4762 | 4.3% |
|  | Unknown primary carcinoma | 4607 | 4.2% |
|  | Urothelial carcinoma | 3236 | 2.9% |
|  | Cholangiocarcinoma | 2901 | 2.6% |
|  | Melanoma | 2743 | 2.5% |
|  | Glioma | 2350 | 2.1% |
|  | Head and neck squamous cell carcinoma | 1787 | 1.6% |
|  | Uveal melanoma | 142 | 0.1% |
| Patient sex | Sex available | 97686 | 89.1% |
|  | Female | 51280 | 52.5% |
|  | Male | 46399 | 47.5% |
|  | Unknown | 7 | 0.0% |
| Patient age | Age available | 97700 | 89.1% |
|  | 0-14 years | 401 | 0.4% |
|  | 15-39 years | 3749 | 3.8% |
|  | 40-64 years | 38255 | 39.2% |
|  | 65-79 years | 44050 | 45.1% |
|  | 80-89+ years | 11245 | 11.5% |

**Table S2.** **Frequencies of prognostic marker findings for patients with select common cancer types.**

| **Cancer Type** | **Prognostic Marker** | **Frequency** | **Percentage** |
| --- | --- | --- | --- |
| Non-small cell lung cancer | | 22152 | 100.0% |
|  | *KRAS* activating mutation | 4084 | 18.4% |
| Colorectal cancer |  | 13193 | 100.0% |
|  | *BRAF* V600E mutation | 969 | 7.3% |
|  | MSI-high | 661 | 5.0% |
| Breast cancer |  | 11016 | 100.0% |
|  | *BRCA* alteration | 1032 | 9.4% |
|  | *BRCA2* alteration | 608 | 5.5% |
|  | *BRCA1* alteration | 438 | 4.0% |
|  | *ERBB2* amplification | 832 | 7.6% |
| Prostate cancer |  | 6513 | 100.0% |
|  | *PTEN* and *TP53* alteration | 1107 | 17.0% |
|  | *RB1* and *TP53* alteration | 294 | 4.5% |
|  | *PTEN* and *RB1* alteration | 200 | 3.1% |
|  | *PTEN* and *RB1* and *TP53* alteration | 137 | 2.1% |
|  | *BRCA* alteration | 660 | 10.1% |
|  | *BRCA2* alteration | 587 | 9.0% |
|  | *BRCA1* alteration | 80 | 1.2% |
| Cholangiocarcinoma |  | 2901 | 100.0% |
|  | *FGFR2* fusion/rearrangement | 235 | 8.1% |
| Melanoma |  | 2743 | 100.0% |
|  | *NRAS* activating mutation | 660 | 24.1% |
| Glioma |  | 2350 | 100.0% |
|  | *TERT* promoter mutation | 1319 | 56.1% |
|  | *TP53* alteration | 1054 | 44.9% |
|  | *IDH1* R132 mutation | 446 | 19.0% |
|  | *ATRX* alteration | 379 | 16.1% |
|  | *H3-3A* K27M mutation | 102 | 4.3% |
|  | *IDH2* R140/R172 mutation | 4 | 0.2% |
| Head and neck squamous cell carcinoma | | 1787 | 100.0% |
|  | *EGFR* amplification | 127 | 7.1% |
| Uveal melanoma |  | 142 | 100.0% |
|  | *BAP1* alteration | 84 | 59.2% |
|  | *SF3B1* alteration | 34 | 23.9% |

**Table S3.** **Frequencies of diagnostic marker findings for patients with select common cancer types.**

| **Cancer Type** | **Diagnostic Marker** | **Frequency** | **Percentage** |
| --- | --- | --- | --- |
| Breast cancer |  | 11016 | 100.0% |
|  | *CDH1* alteration | 861 | 7.8% |
|  | *ERBB2* amplification | 832 | 7.6% |
| Gastroesophageal cancer |  | 4762 | 100.0% |
|  | *CDH1* alteration | 297 | 6.2% |
| Melanoma |  | 2743 | 100.0% |
|  | *GNAQ* R183C/Q209 mutation | 50 | 1.8% |
|  | *GNA11* R183C/Q209 mutation | 49 | 1.8% |
| Glioma |  | 2350 | 100.0% |
|  | *TERT* promoter mutation | 1319 | 56.1% |
|  | *TP53* alteration | 1054 | 44.9% |
|  | *EGFR* amplification | 544 | 23.1% |
|  | *CDKN2A/B* loss | 488 | 20.8% |
|  | *IDH1* R132 mutation | 446 | 19.0% |
|  | *ATRX* alteration | 379 | 16.1% |
|  | *H3-3A* mutation | 136 | 5.8% |
|  | *H3-3A* K27M mutation | 102 | 4.3% |
|  | *H3-3A* G34R mutation | 34 | 1.4% |
|  | *BRAF* fusion | 66 | 2.8% |
|  | *KIAA1549-BRAF* fusion | 57 | 2.4% |
|  | *IDH2* R140/R172 mutation | 4 | 0.2% |
| Uveal melanoma |  | 142 | 100.0% |
|  | *GNA11* R183C/Q209 mutation | 70 | 49.3% |
|  | *GNAQ* R183C/Q209 mutation | 63 | 44.4% |

**Table S4. Absolute and relative frequency of reports with clinical trials matched to the patient’s tumor profile based on evidence; shown for all and 10 specific cancer types.**

| **Cancer Type** | **Total Number of Reports** | **Number with Clinical Trials** | **Percentage with Clinical Trials** |
| --- | --- | --- | --- |
| All | 109695 | 97829 | 89.2% |
| Non-small cell lung cancer | 22152 | 21205 | 95.7% |
| Colorectal cancer | 13193 | 11531 | 87.4% |
| Breast cancer | 11016 | 10095 | 91.6% |
| Ovarian cancer | 6999 | 6429 | 91.9% |
| Prostate cancer | 6513 | 5329 | 81.8% |
| Pancreatic adenocarcinoma | 6168 | 6085 | 98.7% |
| Gastroesophageal adenocarcinoma | 4762 | 4063 | 85.3% |
| Unknown primary carcinoma | 4607 | 4068 | 88.3% |
| Urothelial carcinoma | 3236 | 2981 | 92.1% |
| Cholangiocarcinoma | 2901 | 2525 | 87.0% |

**Table S5. Type and frequency of potential resistance alterations in *EGFR*-mutated lung cancer.**

| **Cancer Type** | **Resistance Mechanism** | **Frequency** | **Percentage** |
| --- | --- | --- | --- |
| NSCLC^a^ | N (all *EGFR*-mutated NSCLC^a^ samples analyzed for potential resistance alterations) | 3260 | 100.0% |
|  | On-target resistance mutations | 259 | 7.9% |
|  | *EGFR* T790M mutation | 211 | 6.5% |
|  | *EGFR* osimertinib resistance mutation | 83 | 2.5% |
|  | C797X (all C797S) mutation | 52 | 1.6% |
|  | L718X mutation | 19 | 0.6% |
|  | G724S mutation | 11 | 0.3% |
|  | L792H mutation | 1 | <0.1% |
|  | Off-target resistance alterations |  |  |
|  | Established bypass signaling mechanisms | 396 | 12.1% |
|  | *MET* activation | 162 | 5.0% |
|  | *MET* amplification | 155 | 4.8% |
|  | *MET* activating mutation | 9 | 0.3% |
|  | *BRAF/MEK/RAS* activation | 138 | 4.2% |
|  | *KRAS* amplification | 61 | 1.9% |
|  | *BRAF* fusion/rearrangement | 28 | 0.9% |
|  | *KRAS* mutation | 25 | 0.8% |
|  | *HRAS/NRAS* amplification/mutation | 16 | 0.5% |
|  | *BRAF* V600E mutation | 10 | 0.3% |
|  | *MEK1/2* mutation | 3 | 0.1% |
|  | *ERBB2* activation | 89 | 2.7% |
|  | *ERBB2* amplification | 73 | 2.2% |
|  | *ERBB2* activating mutation | 17 | 0.5% |
|  | Oncogenic fusion | 30 | 0.9% |
|  | *ALK* fusion/rearrangement | 10 | 0.3% |
|  | *ROS1* fusion/rearrangement | 8 | 0.2% |
|  | *FGFR2/3* fusion/rearrangement | 7 | 0.2% |
|  | *RET* fusion | 4 | 0.1% |
|  | *NTRK* fusion | 3 | 0.1% |
|  | Alterations associated with risk of SCLC^b^ transformation |  |  |
|  | *RB1* and *TP53* alteration | 317 | 9.7% |
| SCLC^b^ | N (all SCLC^b^ samples analyzed) | 963 | 100.0% |
|  | Potential SCLC^b^ transformation |  |  |
|  | *EGFR* mutation | 34 | 3.5% |
|  | *EGFR* mutation and *RB1* and *TP53* alteration | 28 | 2.9% |

^a^non-small cell lung cancer, ^b^small cell lung cancer
